# Supplementary material for: Identification of photoperiod-induced specific miRNAs in the adrenal glands of Sunite sheep (Ovis aries)
Source: Front Vet Sci. 2022 Jul 22;9:888207. doi: 10.3389/fvets.2022.888207 (PMC9354845; doi:10.3389/fvets.2022.888207)
Supplement: Supplementary file 1 [file Data_Sheet_1.ZIP › Supplementary file/miRDeep2 file.zip!/miRDeep2 file/Novel_Structure/LP42A1/Novel_233.pdf]

Provisional ID	:	NC_019462.2_12964
Score total	:	2.7
Score for star read(s)	:	-1.3
Score for read counts	:	0
Score for mfe	:	2.4
Score for randfold	:	1.6
Score for cons. seed	:	
Total read count	:	104
Mature read count	:	104
Loop read count	:	0
Star read count	:	0

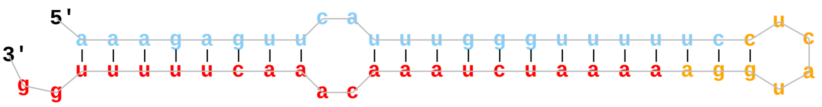

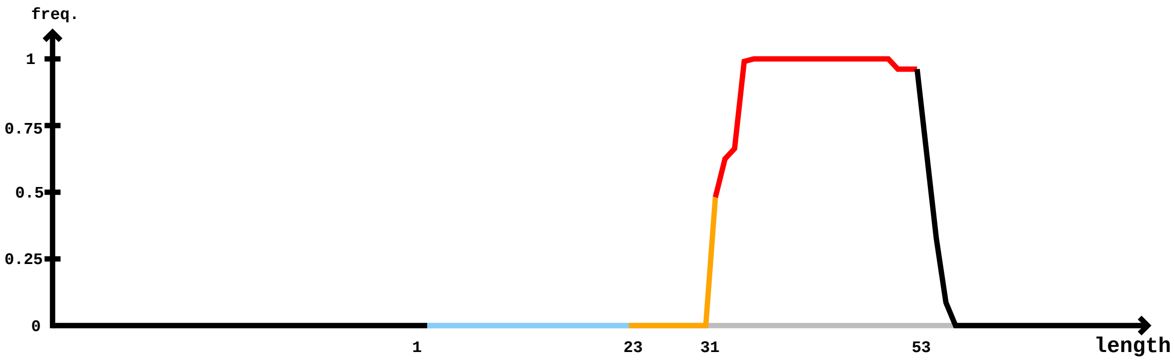

Star Mature

```
5'- gguugaguaguauuccauuguauauauuggguuggccaaagaguucauuuggguuuuuccucauggaaaaaucuaaacaaacuuuuuggccaacccaguauauauacagcau   -3'    exp
    .((((.............(((((((((((((((((((((((((((..(((((((((((((....)))))))))))))..)))))))))))))))))))))))))))))))..    reads       mm          sample
    ...................................................................aaaaucuGaacaaacuuuu..........................    4           1           seq
    ...................................................................aaaaucuGaacaaacuuuuugg.......................    45          1           seq
    ...................................................................aaaaucuGaacaaacuuuuuggc......................    1           1           seq
    ....................................................................aaaucuGaacaaacuuuuugg.......................    14          1           seq
    ....................................................................aaaucuaaacaaacuuuuuggc......................    1           0           seq
    .....................................................................aaucuGaacaaacuuuuuggc......................    4           1           seq
    ......................................................................aucuGaacaaacuuuuuggcc.....................    25          1           seq
    ......................................................................aucuaaacaaacuuuuuggcca....................    1           0           seq
    ......................................................................aucuGaacaaacuuuuuggcca....................    8           1           seq
    .......................................................................ucuGaacaaacuuuuuggc......................    1           1           seq
```
